# How to Test the Association Between Baseline Performance Level and the Modulatory Effects of Non-Invasive Brain Stimulation Techniques

**DOI:** 10.3389/fnhum.2022.920558

**Published:** 2022-06-24

**Authors:** Carlotta Lega, Luigi Cattaneo, Giulio Costantini

**Affiliations:** ^1^Department of Psychology and Milan Center for Neuroscience (NeuroMI), University of Milano-Bicocca, Milan, Italy; ^2^Center for Mind/Brain Sciences (CIMeC), University of Trento, Trento, Italy; ^3^Centre for Medical Sciences (CISMed), University of Trento, Trento, Italy

**Keywords:** brain stimulation, baseline performance, mathematical coupling, regression to the mean, measurement error

## Abstract

Behavioral effects of non-invasive brain stimulation techniques (NIBS) can dramatically change as a function of different factors (e.g., stimulation intensity, timing of stimulation). In this framework, lately there has been a growing interest toward the importance of considering the inter-individual differences in baseline performance and how they are related with behavioral NIBS effects. However, assessing how baseline performance level is associated with behavioral effects of brain stimulation techniques raises up crucial methodological issues. How can we test whether the performance at baseline is predictive of the effects of NIBS, when NIBS effects themselves are estimated with reference to baseline performance? In this perspective article, we discuss the limitations connected to widely used strategies for the analysis of the association between baseline value and NIBS effects, and review solutions to properly address this type of question.

## Introduction

Converging evidence demonstrates that the behavioral effects of transcranial magnetic stimulation (TMS) could dramatically change as a function of different factors, such as stimulation intensity (Moliadze et al., 2003; Abrahamyan et al., 2011, 2015; Silvanto et al., 2017), timing of stimulation (Kammer, 2007; de Graaf et al., 2014; Chiau et al., 2017; Silvanto et al., 2017) and the initial brain “state” when stimulation is applied (Siebner, 2004; Silvanto and Pascual-Leone, 2008; Ruzzoli et al., 2010; Schwarzkopf et al., 2011; Perini et al., 2012; Romei et al., 2016; Silvanto and Cattaneo, 2017). In this framework, there is lately a growing interest toward the importance of considering the inter-individual differences in baseline performance when describing the impact of TMS. Although few studies directly investigated the role of inter-individual differences in determining the behavioral effects of non-invasive brain stimulation techniques (NIBS), there is currently a strong drive to explore how NIBS effects covary with individual characteristics, especially with the performance at baseline (see Silvanto et al., 2018).

A consistent body of independent and recent evidence suggests that baseline performance modulates TMS effects (Schwarzkopf et al., 2011; Painter et al., 2015; Emrich et al., 2017; Juan et al., 2017; Paracampo et al., 2018; Silvanto et al., 2018). Furthermore, also the effects of others brain stimulation techniques, such as transcranial direct current stimulation (tDCS) or transcranial random noise stimulation (tRNS), seem to interact with baseline performance level (Jones and Berryhill, 2012; Tseng et al., 2012; Hsu et al., 2014, 2016; Benwell et al., 2015; Learmonth et al., 2015; Juan et al., 2017; Penton et al., 2017; Schaal et al., 2017; Yang and Banissy, 2017; see also Vergallito et al., 2022 for a recent review). Together these findings have been interpreted as indicative of the importance of adopting an individual differences approach, when describing the effect of NIBS. This is indeed a potentially important issue for brain stimulation studies: Even though at group level a modulation in performance does not emerge, a deeper analysis focusing on individual differences may disclose stimulation effects characterizing specific classes of individuals (Silvanto et al., 2018). According to this view, baseline performance can be seen as an indirect measure of neural excitability that, in interaction with the TMS intensity, contributes to the behavioral outcome (Silvanto and Cattaneo, 2017; Silvanto et al., 2017, 2018). The facilitatory vs. inhibitory effect of TMS as a function of neuronal excitability is a well-established mechanism and it is consistently observed when TMS is applied during a behavioral task following a predictable manipulation of the initial neural state, such as adaptation or priming (see Silvanto et al., 2008 for a review). State-dependent TMS effects in paradigms based on priming/adaptation have been observed in a range of different domains, from number and letter processing (Kadosh et al., 2010; Cattaneo Z. et al., 2010; Renzi et al., 2011) to action observation (Cattaneo, 2010; Cattaneo L. et al., 2010; Jacquet and Avenanti, 2015) and perception of emotion (Mazzoni et al., 2017).

Assessing how baseline performance level (and brain state) determine behavioral effects of brain stimulation techniques is therefore an important question, which raise up crucial methodological issue. How to assess the association between baseline value and subsequent change? Or, in other words, how can we test whether the performance at baseline is predictive of the effect of NIBS? An approach that has been typically used to provide evidence of an association between baseline performance and their changes after the stimulation is the *correlation approach*. This consists in regressing or correlating the magnitude of the induced stimulation effect (which is defined as the performance in the effective TMS/tDCS condition minus the performance in the baseline/Sham condition) with the baseline level of performance (sham stimulation) (Emrich et al., 2017; Penton et al., 2017; Yang and Banissy, 2017; Paracampo et al., 2018; Silvanto et al., 2018; Diana et al., 2021; Wu et al., 2021). Another conceptually similar approach is the *categorization approach* (Tu et al., 2005). It consists in categorizing subjects according to threshold values, such as the median baseline performance (i.e., median-split) and subsequently comparing the effect of NIBS in terms of changes in the behavioral outcome (defined as the active TMS/tDCS condition minus the baseline performance) across the two subgroups (i.e., “low” performers vs. “high” performers) (Tseng et al., 2012; Hsu et al., 2014, 2016; Benwell et al., 2015; Learmonth et al., 2015; Juan et al., 2017; Schaal et al., 2017; Silvanto et al., 2018). However, these approaches are connected to severe biases in estimating the effects. Albeit such biases are well documented (Oldham, 1962; Tu et al., 2005; Chiolero et al., 2013), they have been neglected in several TMS/tDCS studies. We first illustrate biases connected to these methods, and we conclude by discussing techniques that have been proposed to investigate baseline modulatory effects without incurring in such biases.

### Biases of the Correlation Approach

The correlation approach consists in correlating or regressing a baseline with a deviation from the baseline, or equivalently in regressing the deviation from the baseline on the baseline. One issue with this strategy is not taking into account that the estimate of the deviation from a baseline depends on the baseline itself (Oldham, 1962). This issue is known as *mathematical coupling*, and can take place when a correlation is estimated between two variables that share a common source of variation (Blance et al., 2005). Let us denote as *x*_*i*_ the observed baseline performance of the *i*-th individual and as *y*_*i*_ the performance observed after NIBS. The deviation of *i*'s performance from the baseline is computed as *d*_*i*_=*y*_*i*_−*x*_*i*_. The relationship between baseline performance and NIBS effect can be then estimated as the correlation *r*_*d, x*_ = *r*_*y*−*x***,*x***_. We should suspect a mathematical coupling by seeing that *x* contributes to both variables being correlated. Since *x* contributes positively to the first term of the correlation and negatively to the second term, the expected correlation is negative (Spearman, 1913).

A simple numeric example is probably the most effective way to illustrate how dramatic the effects of mathematical coupling can be (Oldham, 1962). We can use the R statistical language (R Core Team, 2021) to generate random data representing the performance of *N* = 50 subjects in the baseline (*x*) and experimental stimulation (*y*) conditions.

**Table:** 

1	set.seed(1)
2	x <- rnorm(50)
3	y <- rnorm(50)
4	t.test(x, y, paired = TRUE)
5	cor(x, y)
6	cor(x, y – x)

**Figure 1:**
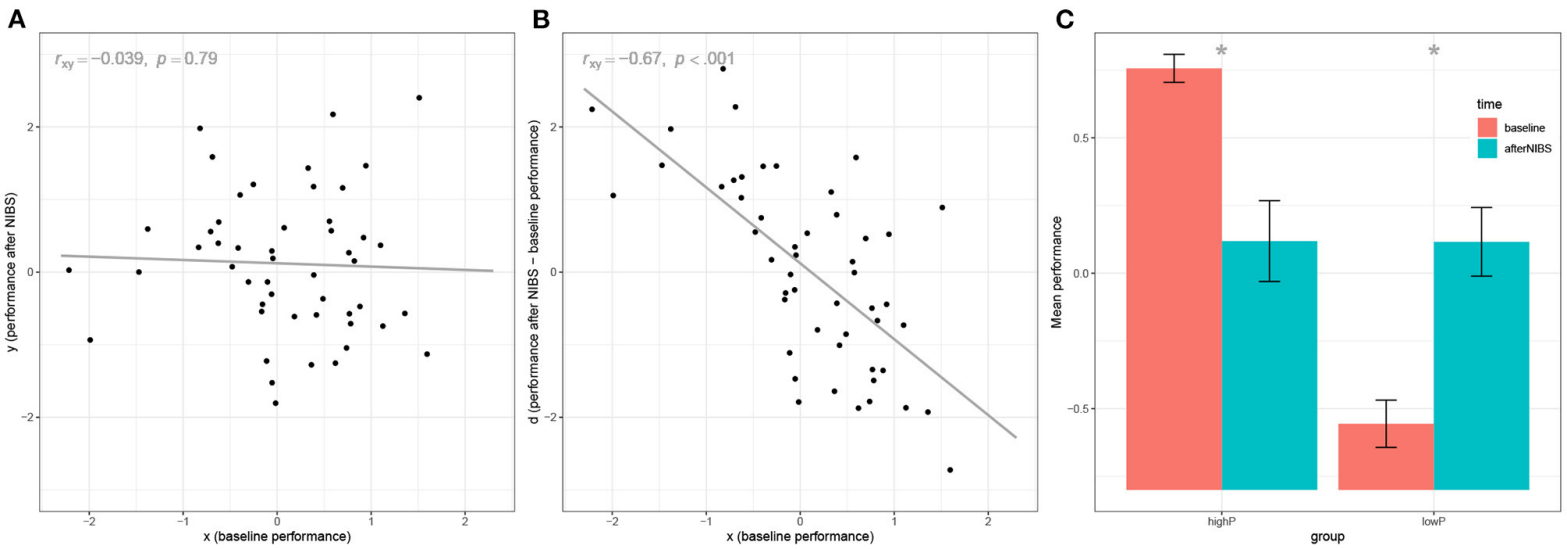
Illustration of mathematical coupling and regression to the mean using a numeric example with *N* = 50 subjects. **(A)** visualizes the relationship between the simulated baseline performance (*x*) and the simulated performance after NIBS (*y*). Data are simulated to be uncorrelated. **(B)** shows the biases in the correlation approach, by visualizing the relationship between the baseline performance (*x*) and the deviation from the baseline after NIBS (*d*). **(C)** shows the biases in the categorization approach: The sample is split into high-performers (*highP*) and low-performers (*lowP*) and the baseline performance is compared with the performance after NIBS. Bars represent ±1 *SE*.

The first line, set.seed(1), serves to fix the random number generation procedure, such that the readers will be able to produce our exact same results on their computers. Lines 2 and 3 actually generate the data at baseline and after NIBS. In this example, all datapoints are independently sampled from a standard normal distribution with μ = 0 and σ = 1. Thus, data come from a population in which there is no relationship between the variables involved and no effect of neurostimulation whatsoever. In short, such data come from a population in which the null hypothesis is true for all parameters of interest. In fact, at line 4, we can test the effect of the NIBS by comparing performance before and after stimulation, and obtain a null result, *t*(49) = −0.09, *p* = 0.93, as it could be expected. Similarly, at line 5, we test the correlation between *x* and *y* and obtain a null result, *r* = −0.039, *p* = 0.79 (Figure 1A). However, at line 6 we test the correlation between the baseline and the deviation from the baseline, and we obtain *r*_*y*−*x, x*_ = −0.67, *p* < 0.001 (Figure 1B). If we repeated the example with a different random *seed*, we would obtain slightly different results each time. On average, it can be demonstrated that our results would converge toward the value $r_{y-x,x}=\;\frac{1}{\sqrt{2}}≅\;0.707$ (Spearman, 1913; Chiolero et al., 2013).^1^ Thus, under the null hypothesis of no relationships and no effect of neurostimulation, a researcher using the correlation approach would expect to find a correlation between the baseline and the deviation that can be considered very large (Cohen, 1988).

Another bias connected to the correlation approach is regression toward the mean due to measurement error at baseline (Nesselroade et al., 1980; Blomqvist, 1987; Tu et al., 2005). Let us now consider the fact that the individual performance is always assessed with a certain degree of error. Variations in observed performance could reflect transient and non-systematic factors (tiredness, distraction, etc.), which introduce noise in the assessment. The observed performance at baseline of the *i*-th subject could be decomposed into *x*_*i*_ = *X*_*i*_+*e*_*xi*_, where the observed performance *x*_*i*_ is given by the sum of the true performance *X*_*i*_ and measurement error *e*_*xi*_. The same holds for the performance after NIBS, *y*_*i*_ = *Y*_*i*_+*e*_*yi*_. A researcher's aim would be to estimate the *true* correlation, which is the correlation involving the true latent performance *r*_*Y*−*X, X*_, but the researcher would typically approximate that value by estimating the correlation involving observed performance, *r*_*y*−*x, x*_. However, the observed difference *y*_*i*_−*x*_*i*_ is equal to *Y*_*i*_−_*X*_*i*_+*eyi*_−*e*_*xi*_. The correlation *r*_*y*−*x, x*_ is thus affected by the measurement error *e*_*xi*_ being present both in the independent and in the dependent variable, with opposite signs. In particular, Blomqvist (1987) has shown that the relationship between the observed correlation (or regression slope) and the true value is *r*_*y*−*x, x*_ = *r*_*Y*−*X, X*_(1−*k*)−*k*, where $k=\frac{\sigma_{e_{x}}^{2}}{\sigma_{x}^{2}}$ is the measurement error, the ratio of the error variance of the observed baseline performance, $\sigma_{e_{x}}^{2}$, to the total variance of the observed baseline performance, σ_*x*_. For example, let us assume a situation in which the true correlation is very low, *r*_*Y*−*X, X*_ = 0.01, and the error variance in performance assessment is 40% (a value that is not uncommon in tasks used in the field, e.g., Fan et al., 2002). The researcher would expect to observe a correlation of *r*_*y*−*x, x*_ = −0.394, which would be regarded as significantly different from zero (*p* < 0.05) on a sample of more than *N* = 25 participants.

Interestingly, both biases lead to the same type of results, which is observing a negative correlation between baseline and change. This is exactly what most of the studies in the field reviewed above reported.

### Biases of the Categorization Approach

The categorization approach consists in categorizing subjects into two groups, one including those with higher baseline performance (e.g., above the median) and the other including the remaining subjects. The deviation in performance between the two groups is then compared (e.g., in using *t*-test or ANOVA). The categorization approach avoids mathematical coupling but is nonetheless affected by regression toward the mean.

As for the correlation approach, an easy way to understand why the categorization approach is problematic is considering what would happen if this was applied under the null hypothesis. Let us define the null hypothesis as the one in which the neurostimulation has no effect whatsoever and the true performance of all participants is the same. For simplicity, let us assume that the true performance takes value zero (i.e., *X*_*i*_ = *Y*_*i*_ = 0, ∀*i*). In this situation, any variance in the observed performance is just measurement noise. Following the typical categorization approach, we would nonetheless perform a median or mean split, relying on the observed baseline performance, and divide participants into high performers (*highP*) and low performers (*lowP*). The observed performance of the *highP* group will thus be always larger than the observed performance of the *lowP* group, but this will not be true for the performance after neurostimulation. Therefore, we will typically observe a performance increase after stimulation for the *lowP* group and a performance decrease for the *highP* group.

This bias can also be easily illustrated by continuing the simple numeric example used above. In particular, lines 7–10 in the code below perform the median split, separating the *x* and *y* variables for those who have better or worse observed baseline performance. Lines 11–12 perform a paired-samples *t*-test to examine changes in performance in the *lowP* group and calculate the effect size *d*_*z*_ (Cohen, 1988; Perugini et al., 2018). Lines 13–14 replicate the analysis for the *highP* group.

**Table:** 

7	x_lowP <- x[x < = median(x)]
8	x_highP <- x[x > median(x)]
9	y_lowP <- y[x < = median(x)]
10	y_highP <- y[x > median(x)]
11	t.test(x_lowP, y_lowP, paired = TRUE)
12	mean(y_lowP – x_lowP)/sd(y_lowP – x_lowP)
13	t.test(x_highP, y_highP, paired = TRUE)
14	mean(y_highP – x_highP)/sd(y_highP – x_highP)

The results of the *t*-test show, as expected, a significant performance improvement for the *lowP* group, *t*(24) = −2.90, *p* = 0.008*, d*_*z*_ = 0.58, as well as a significant decrease in performance for the *highP* group, *t*(24) = 2.89, *p* = 0.008, *d*_*z*_ = −0.58 (Figure 1C). This is of course an example on few randomly generated data points: If one repeated this example with different random data, the effect size would converge toward the values $d_{z}=\pm \sqrt{\frac{1}{\pi -1}}$ (i.e., ±0.68),^2^ an effect that is considered by Cohen above the medium size [i.e., “one large enough to be visible to the naked eye” (Cohen, 1988)], with a positive sign for the *lowP* group and a negative sign for the *highP* group.

### Suggested Approaches

We have shown how a researcher using either the correlation approach or the categorization approach would easily believe to have found a potentially interesting effect, even in a situation in which no effect is present, just because of mathematical coupling and regression to the mean. However, investigating how baseline performance can modulate NIBS effects is a very interesting research question and should not be neglected. Methods for testing such effects have been developed that allow reducing the impact of such systematic biases.

The first method has been proposed by Oldham (Oldham, 1962), and it consists in simply correlating the mean (or, equivalently, the sum) of the performance at baseline and after the stimulation (i.e., $\frac{\text{x}+\text{y}}{2}$) with the performance change (x−y). Albeit this method might appear very similar to the correlation method illustrated above, it can be demonstrated that it gets rid of the mathematical coupling (Tu and Gilthorpe, 2007). This method can be used in any situation in which the correlation approach is used, by simply changing one of the terms.

It has been shown that Oldham's method is equivalent to test a change in variance between *x* and *y* (Tu and Gilthorpe, 2007) and that a differential effect of NIBS according to the baseline implies a change in variance (Chiolero et al., 2013). An alternative test similar to Oldham's method is to directly test the differences between the variances of *x* and *y* (Tu and Gilthorpe, 2007). However, an important limitation of both this and Olhdam's methods is that any factor increasing or decreasing variance after NIBS other than the genuine stimulation effects, namely a change in error variance after NIBS, could lead to spurious conclusions (Tu et al., 2005; Chiolero et al., 2013).

In the above discussion, we have shown that if *x* and *y* are unrelated, the expected correlation between the baseline and the deviation from the baseline is $r_{y-x,x}=\frac{1}{\sqrt{2}}$. Researchers could then wonder whether it would be possible to test the correlation observed in their samples against this value, instead of zero. The issue is slightly complicated by the fact that the expected correlation is not always $r_{y-x,x}=\frac{1}{\sqrt{2}}$, but it depends on the correlation between *x* and *y*. Tu and colleagues (Tu et al., 2005) showed that the correct value can be calculated as $\sqrt{\frac{1-r_{xy}}{2}}$. This method showed performances comparable to the Oldham's method in simulation (Tu et al., 2005). Like Oldham's method, this strategy assumes that error variance in the assessment of performance does not differ before and after NIBS (Tu et al., 2005).

Another method has been proposed by Blomqvist (1987), which corrects the distortion introduced by regression to the mean due to measurement error in baseline performance. This method requires estimating the parameter *k* mentioned above, to recover the true unbiased correlation from the observed correlation or regression slope using the formula^3^ $r_{y-x,x}=\frac{r_{y-x,x}+k}{\left(1-k\right)}$. Parameter *k*, the measurement error, can be estimated as one minus the reliability of the test used for assessing performance (see Parsons et al., 2019 for guidance on how to estimate reliability in cognitive tests), and should be obtained on data independent of those used for the baseline (Tu and Gilthorpe, 2007). A limit of Blomqvsist's method is that it does not correct for regression to the mean due to factors other than measurement error, such as that due to genuine heterogeneity in the responses of patients to treatments (Tu and Gilthorpe, 2007).

Methods based on multilevel linear models have also been suggested to obtain unbiased estimates. In particular, if one has available many repeated measures over time and is interested in estimating whether the (e.g., linear) trend in change over time is related to the baseline, one can employ multilevel linear models and estimate the correlation between random intercept (i.e., the interindividual variance in the baseline performance) and random slope (i.e., the interindividual variance in the deviation from the slope) (Byth and Cox, 2005; Chiolero et al., 2013). This is also possible if only two assessments of performance are available, but estimating such models requires constraining error variance to zero to make the model identified (Blance et al., 2005). When using mixed models, it is crucial to center the predictor variable (i.e., time should be coded as −0.5 if before NIBS and +0.5 if after NIBS, not as 0 and 1), otherwise estimates will be vulnerable to mathematical coupling (Blance et al., 2005). Unlike other methods reviewed, this solution allows testing more elaborated models including also covariates (Blance et al., 2005).

## Conclusions

The main goal of this perspective article was clarifying the main biases connected to widely used methods to examine the association between baseline performance and NIBS effects, reviewing solutions proposed in the literature. In particular, we have shown that mathematical coupling and regression to the mean can have large distorting effects on estimates, leading to extremely biased conclusions even when the null hypothesis is true. We also reviewed several solutions to mitigate such biases. None of the methods reviewed can be considered as the perfect solution, and whether one of such methods is superior to the others is still debated (Hayes, 1988; Tu et al., 2005; Tu and Gilthorpe, 2007; Chiolero et al., 2013). However, any of these methods will be superior to both the correlation and the categorization approaches that have been used in the field of NIBS. In situations in which is difficult to determine which biases are more likely to affect one's estimate, we suggest to apply different methods (e.g., Oldham's and Blomqvist's method), to inspect the results after considering different sources of bias.

We wish to stress that the biases and the solutions reviewed here are not recent findings. Some of them have been known for more than fifty years (Oldham, 1962; Blomqvist, 1987). Furthermore, these biases are not strictly specific to NIBS, but are relevant whenever one is interested in examining the relationships between baseline levels and deviations from such levels. Nonetheless, knowledge of such biases and solutions does not seem to be effectively integrated in the NIBS literature. The present work thus provides a strong contribution to a deeper understanding of the non-linear effects observed in brain stimulation studies (Schwarzkopf et al., 2011; Jones and Berryhill, 2012; Tseng et al., 2012; Hsu et al., 2014; Benwell et al., 2015; Painter et al., 2015; Learmonth et al., 2015; Emrich et al., 2017; Penton et al., 2017; Schaal et al., 2017; Yang and Banissy, 2017; Paracampo et al., 2018; Silvanto et al., 2018), and represents a step forward toward a full exploitation of the potential of brain stimulation techniques.

## Author Contributions

CL, LC, and GC: conceptualization and writing—review and editing. CL and GC: manuscript preparation. All authors contributed to the article and approved the submitted version.

## Conflict of Interest

The authors declare that the research was conducted in the absence of any commercial or financial relationships that could be construed as a potential conflict of interest.

## Publisher's Note

All claims expressed in this article are solely those of the authors and do not necessarily represent those of their affiliated organizations, or those of the publisher, the editors and the reviewers. Any product that may be evaluated in this article, or claim that may be made by its manufacturer, is not guaranteed or endorsed by the publisher.

## Footnotes

^1^If we used an unstandardized regression approach, our results would converge towards a slope *b* = −1 (Blomqvist, 1987), which is equivalent to a correlation of $\frac{1}{\sqrt{2}}$. This can be seen considering that the population variance of *x* and *y* are σ_*x*_ = σ_*y*_ = 1 and their correlation is σ_*xy*_ = 0. The standard deviation of *y*–*x* is thus $\sigma_{y-x}=\sqrt{\sigma_{x}+\sigma_{y}-\sigma_{xy}}=\sqrt{2}.$ The regression slope *b*_*y*−*x, x*_ can be standardized with the formula $r_{y-x,x}=b_{y-x,x}\frac{\sigma_{x}}{\sigma_{y-x}}=\frac{1}{\sqrt{2}}$.

^2^This value can be obtained by considering that *y*_*lowP*_ and *y*_*highP*_ have been sampled from a standard normal distribution. Since the median of a standard normal distribution is zero, *x*_*highP*_ and *x*_*lowP*_ follow a half-normal distribution, with mean *M* = $\pm \sqrt{\frac{2}{\pi }}$ and SD = $\sqrt{\frac{\pi -2}{\pi }}$ (Olmos et al., 2012). The expected value of the effect size *d*_*z*_ can then be calculated as the mean of the differences between *y*_*lowP*_−*x*_*lowP*_ or *y*_*highP*_−*x*_*highP*_, which are respectively $+\sqrt{\frac{2}{\pi }}\;and-\sqrt{\frac{2}{\pi }}$, divided by the standard deviation of such differences, which is in both cases $\sqrt{\frac{2\pi -2}{\pi }}$. The expected effect size is then given by $d_{z}=\pm \sqrt{\frac{\frac{2}{\pi }}{\frac{2\pi -2}{\pi }}}\text{=}\sqrt{\frac{1}{\pi -1}}$.

^3^Notice that Blomqvist (1987) reports a slightly different formula from those used here, in which *k* is subtracted and not added from the observed correlation/regression slope. This is because in some fields the deviation from baseline is calculated as *y*–*x* (i.e., improvement from baseline), in other fields the same change is calculated as *x*–*y* (i.e., deterioration from baseline), thus implying a change in sign.
